# Supplementary figures and images for: SIRT4 Controls Macrophage Function and Wound Healing through Control of Protein Itaconylation in Mice
Source: bioRxiv. 2025 May 13:2025.05.12.653532. Preprint. [Version 1] doi: 10.1101/2025.05.12.653532 (PMC12132189; doi:10.1101/2025.05.12.653532)

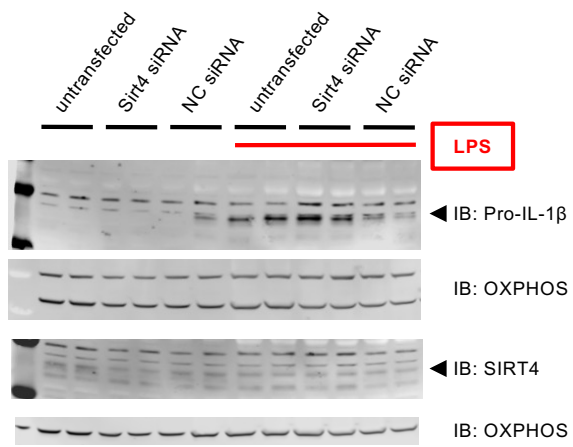

**Supplemental Figure 1 (Corresponds to Fig. 2)**

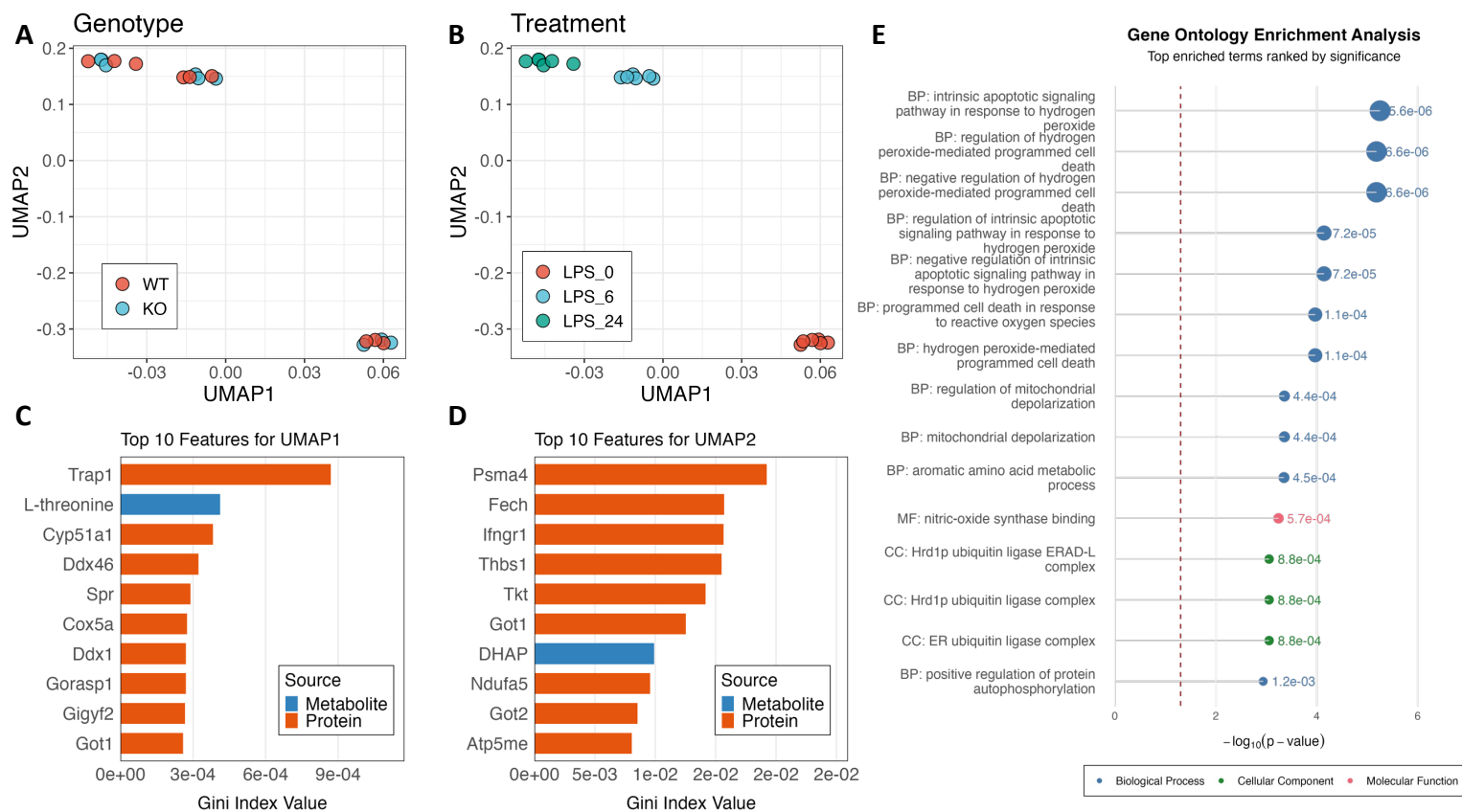

**Supplemental Figure 2 (Corresponds to Fig. 3)**

Supplement: Supplement 1 — Supplemental Figure 1. RAW264.7 cells transfected with SIRT4 siRNA or negative control siRNA for 3 days were stimulated with LPS (500ng/ml) for 24h. IL-1β production was measured by Western Blotting. Supplemental Figure 2. Multi-omic integration of macrophage responses to LPS treatment using GAUDI. A-B. UMAP visualization of integrated metabolomic, proteomic, and itaconylated peptide data, with points representing individual samples colored by genotype (WT vs. KO) (A) or LPS treatment duration (0, 6, or 24 hours) (B). The UMAP space reveals that LPS treatment duration dominates the clustering pattern, with distinct grouping of LPS_0 samples (right clusters) separating from LPS_6 and LPS_24 samples (left clusters). C-D. Top 10 features contributing to UMAP1 (C) and UMAP2 (D) dimensions based on Gini index values from the Random Forest model. Features are color-coded by source (blue: metabolites, orange: proteins). E. Gene Ontology enrichment analysis of features contributing to the integrated GAUDI model shows significant enrichment of hydrogen peroxide-mediated programmed cell death pathways, mitochondrial depolarization processes, and ubiquitin ligase complex activity. These biological processes align with known LPS-induced inflammatory responses and oxidative stress pathways in macrophages, explaining the dominant effect of LPS treatment observed in the clustering patterns. [file media-1.pdf]
