# Supplementary material for: SIRT4 Controls Macrophage Function and Wound Healing through Control of Protein Itaconylation in Mice
Source: bioRxiv. 2025 May 13:2025.05.12.653532. Preprint. [Version 1] doi: 10.1101/2025.05.12.653532 (PMC12132189; doi:10.1101/2025.05.12.653532)
Supplement: Supplement 4 [file media-4.pptx]

## Slide 1
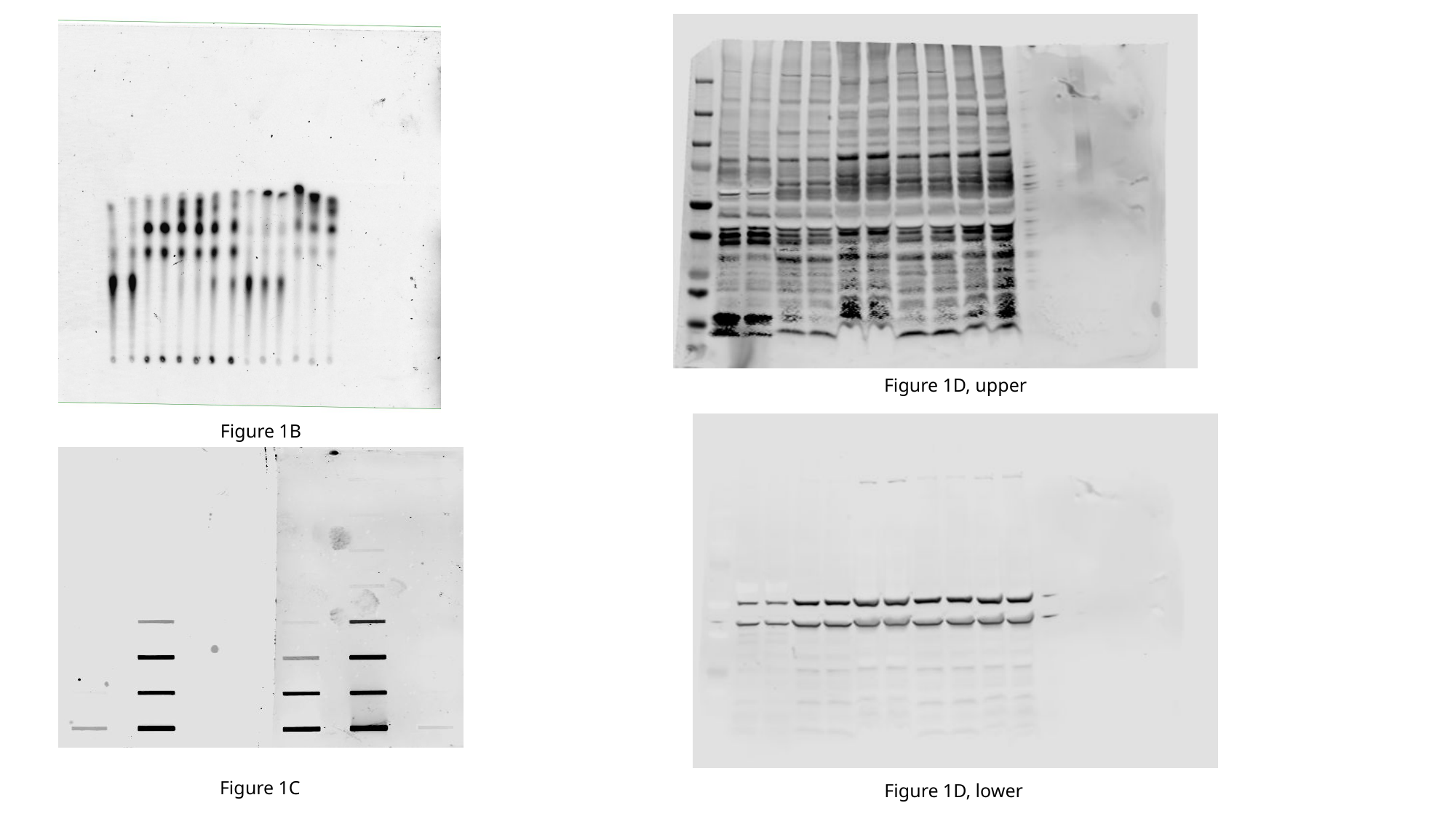

Figure 1D, upper
Figure 1B
Figure 1C
Figure 1D, lower

## Slide 2
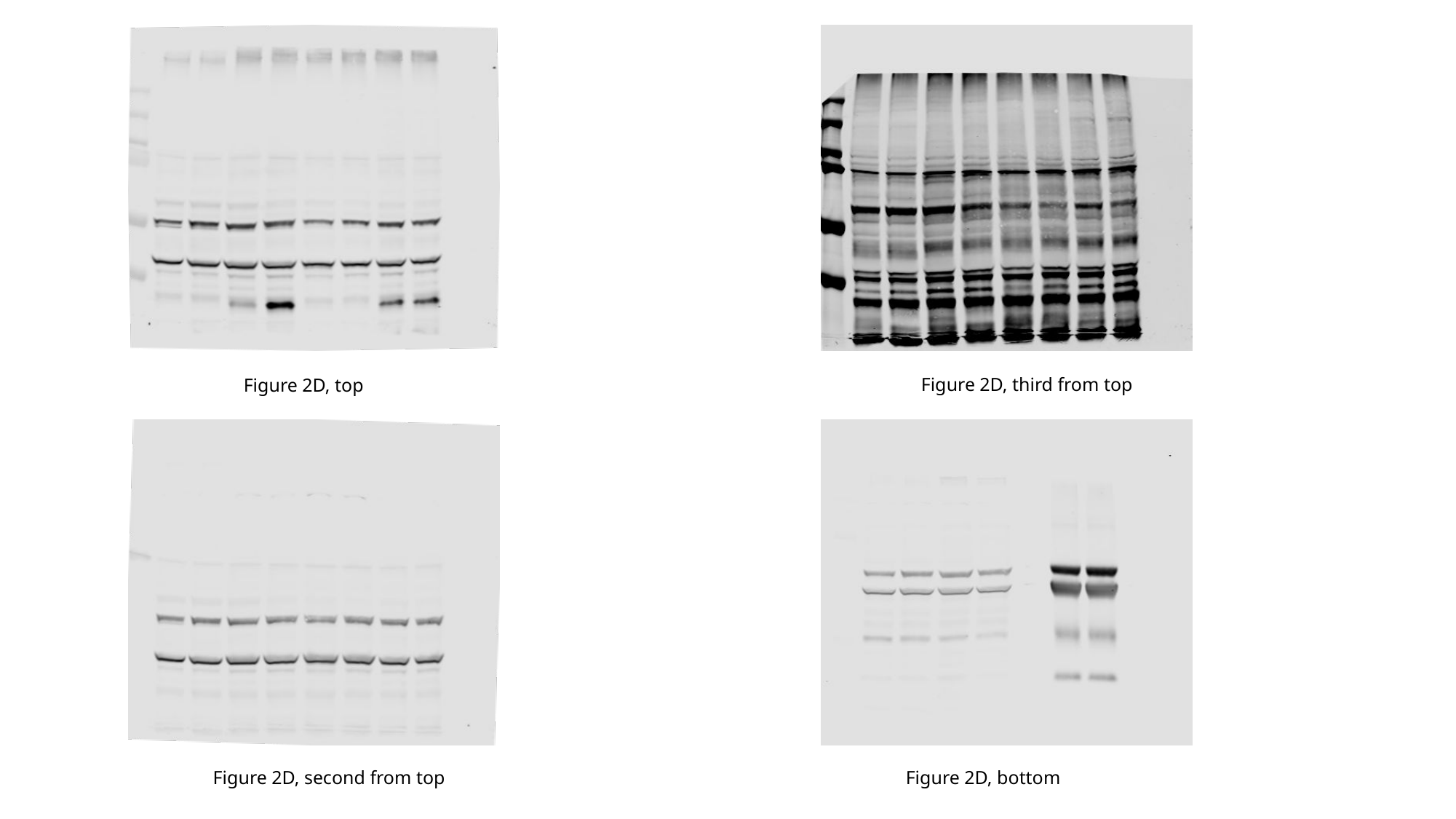

Figure 2D, third from top
Figure 2D, top
Figure 2D, second from top
Figure 2D, bottom

## Slide 3
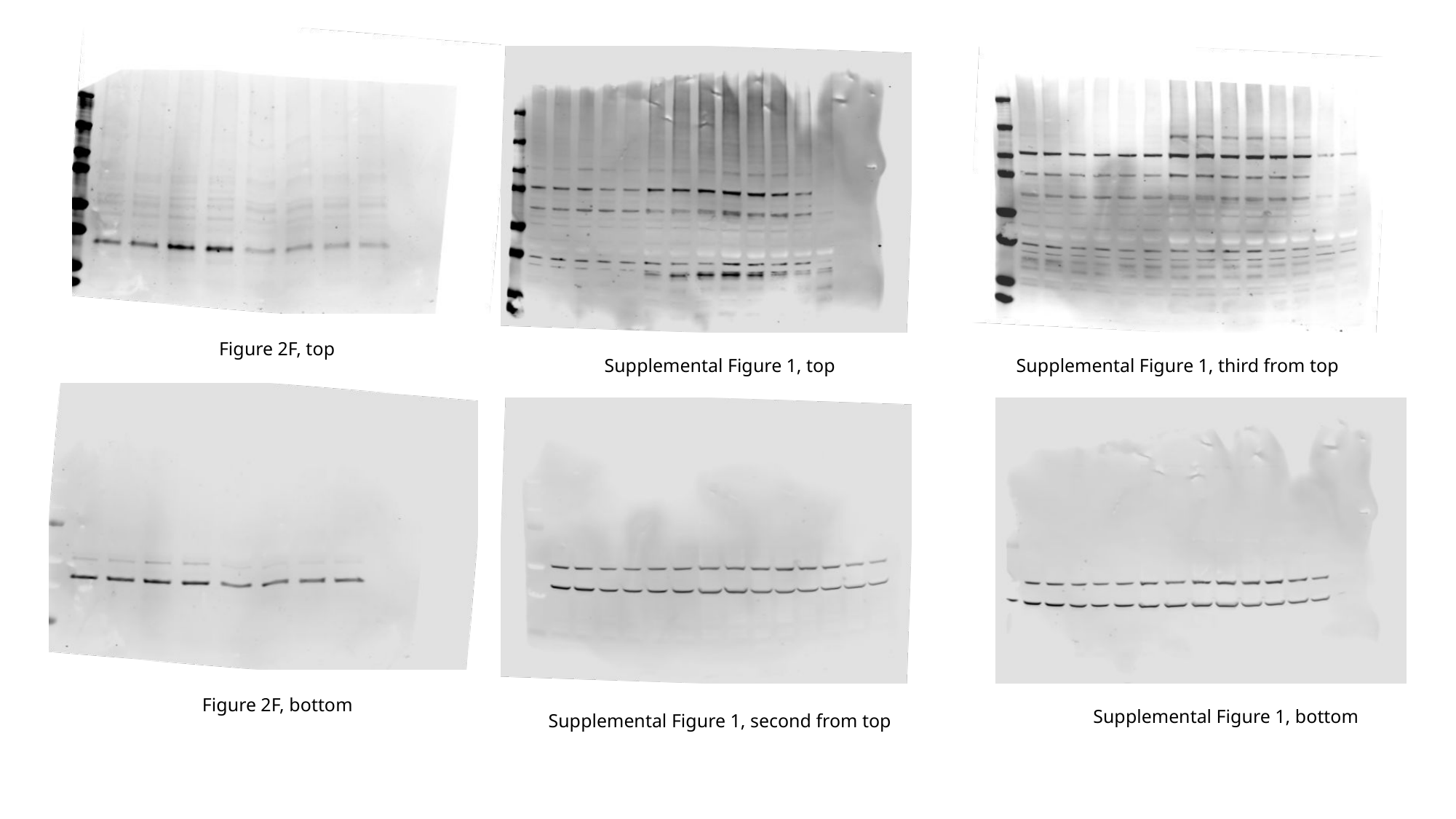

Figure 2F, top
Supplemental Figure 1, third from top
Supplemental Figure 1, top
Figure 2F, bottom
Supplemental Figure 1, bottom
Supplemental Figure 1, second from top
